# Dynamic Path Planning for Unmanned Autonomous Vehicles Based on CAS-UNet and Graph Neural Networks

**DOI:** 10.3390/s25144283

**Published:** 2025-07-09

**Authors:** Yuchu Ji, Rentong Sun, Yang Wang, Zijian Zhu, Zhenghao Liu

**Affiliations:** College of Electronic Information and Automation, Civil Aviation University of China, Tianjin 300300, China; 2023022213@cauc.edu.cn (R.S.); yangwang@cauc.edu.cn (Y.W.); 2024022169@cauc.edu.cn (Z.Z.); 2024022192@cauc.edu.cn (Z.L.)

**Keywords:** path planning, graph neural network, attention mechanisms, multi-unmanned autonomous vehicles

## Abstract

This paper proposes a deeply integrated model called CAS-GNN, aiming to solve the collaborative path-planning problem for multi-agent vehicles operating in dynamic environments. Our proposed model integrates CAS-UNet and Graph Neural Network (GNN), and, by introducing a dynamic edge enhancement module and a dynamic edge weight update module, it improves the accuracy of obstacle boundary recognition in complex scenarios and adaptively changes the influence of different edges during the information transmission process. We generate data through online trajectory optimization to enhance the model’s adaptability to dynamic environments. Simulation results show that our proposed CAS-GNN model has good performance in path planning. In a dynamic scenario involving six vehicles, our model achieved a success rate of 92.8%, a collision rate of 0.0836%, and a trajectory efficiency of 64%. Compared with the traditional A-GNN model, our proposed CAS-GNN model improves the planning success rate by 2.7% and the trajectory efficiency by 8%, while reducing the collision rate by 23%.

## 1. Introduction

Path planning plays an important role in intelligent transportation systems [1], which makes it a crucial aspect of autonomous driving technology. With the development of autonomous driving technology, research on single-vehicle path planning is gradually becoming more mature. Early research on traditional single-vehicle planning focused on using conventional algorithms to identify optimal or sub-optimal paths in known environments. Among the classic algorithms in the path-planning field, the A* algorithm has demonstrated excellent performance on rasterized maps [2]; it is a classic algorithm for solving single-vehicle planning problems and is especially suitable for finding the shortest path in a static environment, with a high success rate and efficiency. On this basis, references [3,4,5] propose improved A* algorithms to address the issues of low exploration efficiency, excessive turns, and rough paths of the A* algorithm, respectively.

Sampling-based searching algorithms, such as the Rapidly-exploring Random Trees (RRT) algorithm and the RRT* algorithm, have also been used to solve the single-vehicle path planning problem [6,7,8]. These sampling-based searching algorithms can rapidly generate candidate paths in high-dimensional complex spaces and gradually converge through optimization. Based on this, references [9,10,11] propose improved RRT* algorithms to address the slow convergence speed of the RRT* algorithm.

Reference [12] proposed a path planning algorithm based on Model Predictive Control (MPC), which considers the vehicle dynamics model and realizes continuous constraints in path planning. In reference [13], a metaheuristic algorithm was used to solve the path planning problem. In the early research, metaheuristic algorithms included fuzzy logic algorithms [14], simulated annealing algorithms [15], etc. Since then, they have expanded to encompass advanced methods such as neural networks [16], genetic algorithms [17], ant colony optimization [18], etc. Metaheuristic algorithms possess strong generalization ability and can adapt to various environments.

With the development of multi-vehicle collaboration technology, multi-unmanned vehicle systems (MUV) have been gradually applied in complex scenarios such as logistics and urban transportation [19]. Thus, the multi-vehicle routing problem has attracted the attention of researchers. Multi-vehicle path planning involves a tradeoff between local obstacle avoidance for each vehicle and overall collaborative efficiency. In recent years, researchers have proposed four kind of algorithms, i.e., the optimization-based planning algorithm [20], sampling-based search algorithm [21,22,23], deep reinforcement learning-based algorithm [24,25,26], and hybrid architecture algorithm. They are all considered to solve the multi-vehicle path planning problem.

The optimization-based algorithm formulates objective functions which include path smoothness, energy consumption, and safe obstacle-avoidance distances. It also establishes constraints, such as vehicle dynamic limitations and collision avoidance. The algorithm then employs mathematical optimization algorithms to determine the optimal path. Wang et al. proposed an MPC path planning and control model [20] which can adaptively adjust the obstacle avoidance weight, thereby solving the problems of easy planning failure and poor obstacle avoidance performance of MPC caused by changes in external parameters.

Sample-based search algorithms identify optimal paths by generating and evaluating candidate routes through a configuration space. These algorithms employ random or heuristic strategies to create samples and then use cost evaluation to select the best solution. Representative algorithms include Rapidly-exploring Random Tree (RRT*) and its improved versions (e.g., Dynamic RRT*, and algorithms derived from A*). Zhao et al. proposed the Dynamic RRT algorithm [21]. They constructed a heuristic sampling subset through path length estimation and decomposed the path optimization problem by integrating the concept of dynamic programming. This algorithm balances convergence speed and path length when navigating environments with randomly distributed obstacles. Researchers have studied conflict detection and resolution strategies to more effectively address conflict problems in multi-vehicle path planning. Time-window-based conflict detection algorithms [22] can predict potential collision risks of vehicles at future time points in real time. The Conflict-Based Search (CBS) algorithm [23] improves problem-solving efficiency by decoupling path planning from conflict resolution in a hierarchical manner.

The hybrid architecture algorithm uses optimization techniques to ensure that vehicle dynamics and safety constraints are strictly enforced during the planning phase. Xu et al. proposed a hybrid planner [27]. The V-Hybrid A* algorithm was employed for global path searches. The optimization algorithm was then used to refine trajectories. This algorithm generated safe, efficient, and smooth cooperative trajectories for multiple vehicles within an unstructured conflict area.

Neural networks improve a system’s ability to adapt to dynamic and unstructured environments. This capability enables the system to better adapt to dynamic and unstructured environments. Li et al. proposed a geometric GNN (GeoGNN) [28] that allows each robot to process sensory data from its neighbors. This model can achieve path planning without a global map and significantly improves path efficiency in complex scenarios. Among these neural network approaches, attention-based Graph Neural Networks (GNNs) show remarkable value in multi-vehicle path planning. GNN models represent each vehicle and its surrounding environment as a graph structure, where the edges represent interactions among vehicles. The network automatically learns the priority of interactions among different vehicles by using an attention mechanism. Liu et al. proposed a trajectory prediction framework based on a multi-agent, multi-modal graph attention isomorphic network (GAIN) [29] to effectively understand and aggregate long-term interactions among agents. Shi et al. created the UniMP model [30], which uses a graph transformer to effectively propagate information throughout the network. Ma et al. proposed a graph neural network A-GNN [31] based on the U-Net architecture and attention mechanism, which achieved multi-vehicle predictive navigation control.

In practical applications, multi-vehicle path planning often integrates multi-source data, including vision, LiDAR, and map information. It also needs a unified decision-making framework based on such data. Existing multi-vehicle dynamic path planning approaches have several problems and limitations. Traditional algorithms relying on searches or optimization perform well in single-vehicle planning scenarios. However, when dealing with multi-vehicle collaboration and dynamic environments, they struggle to balance global optimality, real-time responsiveness, and vehicle dynamics constraints. Metaheuristic algorithms typically suffer from slow convergence and result in instability [32]. Deep reinforcement learning-based algorithms demand substantial amounts of training data and are susceptible to fluctuations during the training process [33], thereby yielding less-than-optimal practical outcomes. Furthermore, current algorithms still show relatively low planning success rates and trajectory efficiency in multi-vehicle, unconstrained scenarios. Their collision avoidance rates also need further enhancement.

In addressing the aforementioned problems, this paper introduces a dynamic path planning model for multiple unmanned autonomous vehicles. This method integrates CAS-UNet [34] with Graph Neural Networks (GNNs) and is referred to as CAS-GNN. Building upon this foundation, we incorporate dynamic edge enhancement to improve the accuracy of obstacle boundary recognition using the CAS-GNN model in complex scenarios. Additionally, the model uses a dual-channel feature interaction strategy coupled with a dynamic edge weight generation mechanism. We use online trajectory optimization to generate training data, strengthening the model’s generalization capability in intricate obstacle avoidance situations. Our simulation results indicate that, compared to the conventional A-GNN model, the proposed CAS-GNN model achieves a success rate of 92.8% in path planning across six vehicle dynamics scenarios. Additionally, it reduces the collision rate by 23% and improves trajectory efficiency by 8%. This model significantly improves collaborative decision-making capabilities among multi-agent vehicles operating in dynamic and complex environments. By leveraging a GNN constructed with an attention mechanism based on the CAS-UNet model, we demonstrate improved performance in dynamic path planning for multiple vehicles within unconstrained scenarios.

## 2. Problem Model

In this paper, we assume an unconstrained environment without traffic rules or routes, which includes N vehicles and N obstacles. The *i*-th vehicle and the *i*-th obstacle nodes are represented as $z_{i_{v\;}}^{0}=[x,y,\theta ,v,\hat{x},\hat{y},\hat{\theta },0{]}^{T}\;and\;z_{i_{o\;}}^{0}=[x,y,\theta ,0,x,y,\theta ,r{]}^{T}$, respectively, where x,y are the initial coordinates, θ is the initial orientation, v is the vehicle’s speed, $\hat{x}$, $\hat{y}$ are the target coordinates of the vehicle, and r is the radius of the obstacle. Since the obstacles are stationary, the parameter v of the obstacle is set as 0, and the target coordinate of the obstacle is set as x,y. The topological relationship of these nodes are constructed with a hybrid connection mode. The goal of this paper is to establish a Graph Neural Network (GNN) model based on the CAS-UNet architecture with an attention mechanism to safely and effectively control multiple vehicles, allowing them to reach their target positions in an unconstrained environment.

The vehicle model in this paper is based on the Kinematic Bicycle Model. We assume that the vehicle is a rigid body moving on a two-dimensional plane; the steering radius and the nonlinear characteristics of the tires are ignored. From time T to T + 1, the motion equations can be described as:

(1) $$\begin{array}{c} x_{t+1}=x_{t}+v_{t}\cdot \cos\left(\theta_{t}\right)\cdot \Delta t\\ y_{t+1}=y_{t}+v_{t}\cdot \sin\left(\theta_{t}\right)\cdot \Delta t\\ \theta_{t+1}=\theta_{t}+v_{t}\cdot \tan\left(\varphi_{t}\right)\cdot \gamma \cdot \Delta t\\ v_{t+1}=\beta \cdot v_{t}+p\cdot \Delta t \end{array}$$

where, φ is the steering angle of the vehicle’s front wheels, p represents the vehicle’s acceleration, Δt is the time step, and β and γ are adjustable parameters representing speed damping and steering response coefficients, respectively.

Based on Equation (1), two objectives should be achieved during the dynamic path planning process, i.e., guiding the vehicle to the target position, and preventing collisions with other vehicles or obstacles. Let $C_{\mathrm{coll}\_\mathrm{obs}}$ and $C_{\mathrm{coll}\_\mathrm{veh}}$ be the cost of a vehicle to obstacle collision and the cost of a vehicle to vehicle collision, respectively, which can be denoted as:

(2) $$\begin{array}{cc} C_{coll\_obs} & =\sum_{t=1}^{H}\;\sum_{i=1}^{N_{v}}\;\sum_{j=1}^{N_{o}}\;\left[\frac{1}{{\left\|X_{t}^{\left(i\right)}-X^{\left(j\right)}\right\|}_{2}-r^{\left(j\right)}}\right.\\ & \left.-\frac{1}{r_{mar\_obs\;}}\right]\cdot \Pi_{obs}^{i,j}\cdot w_{col\_obs\;}\\ \Pi_{obs}^{i,j} & =\left\{\begin{array}{cc} 1 & \left.\left({\left\|X_{t}^{\left(i\right)}-X^{\left(j\right)}\right\|}_{2}-r^{\left(j\right)}-r_{mar\_obs\;}\right)<0\right)\\ 0 & \;otherwise\; \end{array}\right. \end{array}$$

(3) $$\begin{array}{cc} C_{coll\_veh} & =\sum_{t=1}^{H}\;\sum_{i=1}^{N_{v}-1}\;\sum_{j=i+1}^{N_{v}}\;\left[\frac{1}{{\left\|X_{t}^{\left(i\right)}-X_{t}^{\left(j\right)}\right\|}_{2}}\right.\\ & \left.-\frac{1}{r_{mar\_veh}}\right]\cdot \Pi_{veh}^{i,j}\cdot w_{col\_veh}\\ \Pi_{veh}^{i,j} & =\left\{\begin{array}{cc} 1 & \left.\left({\left\|X_{t}^{\left(i\right)}-X_{t}^{\left(j\right)}\right\|}_{2}-r_{mar\_veh\;}\right)<0\right)\\ 0 & otherwise \end{array}\right. \end{array}$$

where H is the predicted time step, and X is the position vector ${\left[x,y\right]}^{T}$, where $X_{t}^{\left(i\right)}$ represents the position vector of the i-th vehicle at time t, $X^{\left(j\right)}$ represents the position vector of the j-th obstacle in the scene, $r^{\left(j\right)}$ represents the radius of the j-th obstacle, and $r_{\mathrm{mar}\_\mathrm{obs}\;}$ represents the safety distance of the obstacle, $\Pi_{\mathrm{obs}\;}^{i,j}$ is the indicator function showing whether the i-th vehicle and the j-th obstacle collide, with a value of 1 if a collision occurs, and 0 otherwise, and $w_{\mathrm{col}\_\mathrm{obs}\;}$ represents the weight coefficient of the obstacle collision cost. Let $C_{\mathrm{tar}\;}$ be the target cost to penalize the distance between the current position and the target position of vehicles, which can be denoted as:

(4) $$\begin{array}{cc} C_{tar} & =\sum_{t=1}^{H}\;\sum_{i=1}^{N_{v}}\;{\left\|X_{t}^{\left(i\right)}-X_{target\;}^{\left(i\right)}\right\|}_{2}\cdot w_{pos}\\ & +{\left\|\theta_{t}^{\left(i\right)}-\theta_{tar}^{\left(i\right)}\right\|}_{2}\cdot w_{orient} \end{array}$$

where $X_{\mathrm{target}\;}^{\left(i\right)}$ represents the target position vector of the *i*-th vehicle, $\theta_{t}^{\left(i\right)}$ represents the direction angle of the i-th vehicle at time t, $\theta_{\mathrm{tar}\;}^{\left(i\right)}$ represents the target direction angle of the i-th vehicle, $w_{\mathrm{pos}\;}$ represents the weight coefficient for position error, and $w_{\mathrm{orient}\;}$ represents the weight coefficient for angle error.

Finally, we use the Sequential Least Squares Programming (SLSQP) algorithm to iteratively search for the minimum value of the objective function under a given constraint.

(5) $$\underset{p,\varphi }{min}\;\left[C_{tar}+C_{coll\_obs\;}+C_{coll\_veh\;}\right]$$

Our model uses two core constraints, $C_{\mathrm{coll}\_\mathrm{veh}\;}$ and $C_{\mathrm{coll}\_\mathrm{obs}\;}$, to achieve effective collision avoidance. Specifically, $C_{\mathrm{coll}\_\mathrm{veh}\;}$ is responsible for maintaining a safe distance between vehicles, while $C_{\mathrm{coll}\_\mathrm{obs}\;}$ is used to maintain a safe distance between vehicles and obstacles. In addition, we introduce the $C_{tar}$ constraint, which guides each vehicle to approach its preset target position and orientation as closely as possible. The trajectory smoothness is optimized by imposing constraints on the vehicle’s steering angle and acceleration.

## 3. CAS-GNN Path Planning

We propose a graph neural network model named CAS-GNN. This model guides each vehicle to its destination while preventing collisions with other vehicles and obstacles.

**Figure 1:**
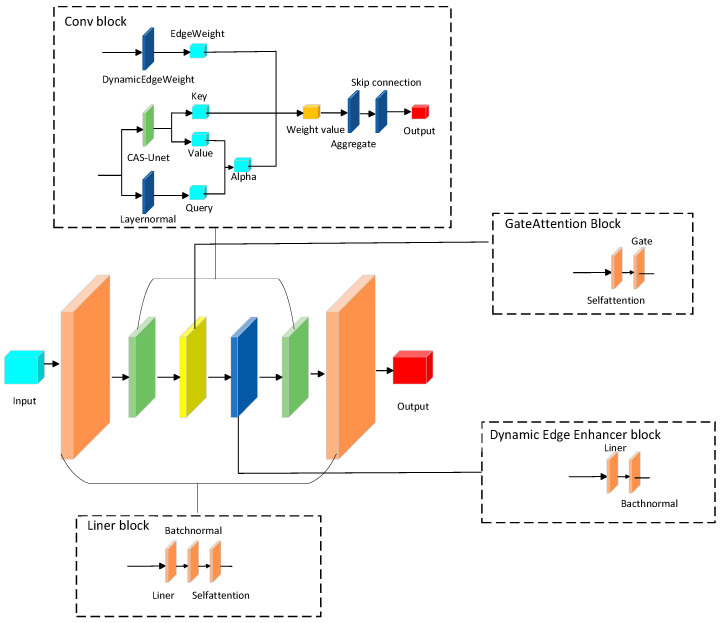
CAS-GNN model.

The CAS-GNN model integrates the CAS-UNet architecture, which enhances the network’s adaptability and generalization capabilities. This architecture includes an attention gate module, a cross-channel attention mechanism, and a dynamic edge weight generation module. Compared to the traditional U-Net, our model demonstrates greater flexibility and expressiveness when processing graph data. The model synergizes graph neural networks, attention mechanisms, and physical constraints to facilitate dynamic scene modeling and prediction. The proposed CAS-GNN model is illustrated in Figure 1.

In this paper, we utilize a dynamic heterogeneous graph to model the traffic scene. The eight-dimensional input node features of each node, denoted as $z_{i}^{0}$, are transformed into higher-dimensional latent vectors $z_{i}^{1}$ through a linear transformation followed by an activation function (ReLU). This process can be expressed as:

(6) $$z_{i}^{1}=ReLU\left(W_{1}z_{i}^{0}\right)\in R^{d_{1}},W_{1}\in R^{d_{1}\times 8}$$

where $W_{1}\in R^{d_{1}\times 8}$ is a trainable weight matrix.

Our proposed CAS-GNN model comprises L residual graph layers. Each layer incorporates four essential modules: graph convolution, gated multi-head attention, dynamic edge enhancement, and residual feature fusion. Within these layers, nodes employ an attention-based mechanism to effectively capture information from neighboring entities. The inclusion of residual connections within the framework facilitates the efficient transfer of prior information, thereby enabling the effective integration of multimodal data.

### 3.1. Graph Convolutional Layer

**Figure 2:**
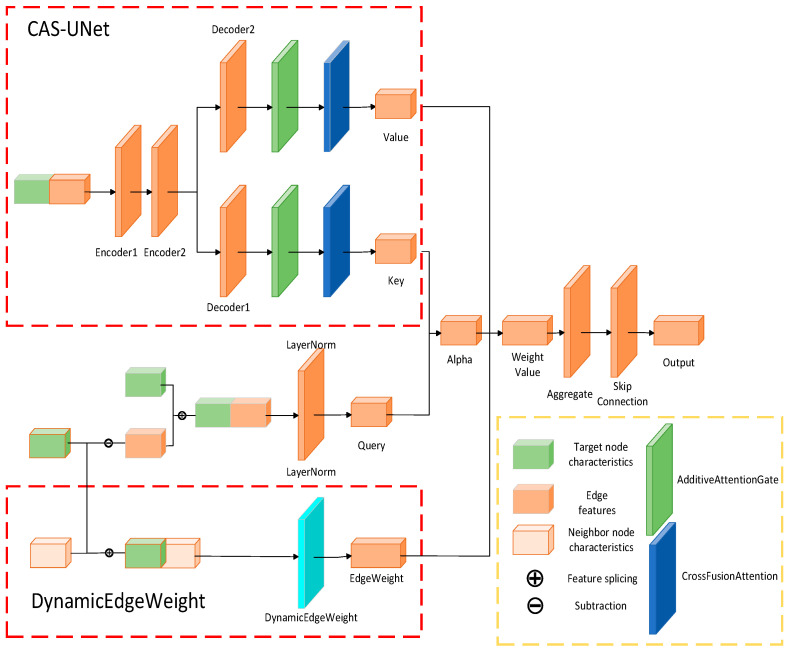
The Graph Convolution Block Structure.

The graph convolutional layer is alternately composed of graph convolution blocks and self-attention layers. The graph convolution block consists of two parts: the CAS-UNet module and the dynamic edge weight update module, as shown in Figure 2.

The graph convolution block extracts information from neighboring nodes while integrating edge weights and node features. This process enhances the capability of feature representation through the improved CAS-UNet structure, ultimately yielding updated features for the nodes. The procedure for convoluting blocks is outlined as follows.

1. Calculate dynamic edge weights.

For the edge (i,j), the dynamic edge weight $e_{ij}$ can be calculated as

(7) $$e_{ij}=\sigma \left(f_{edge\;}\left(z_{i}^{1},z_{j}^{1}\right)\right)$$

where $f_{\mathrm{edge}\;}$ is a multilayer perceptron (Multilayer, MLP), which consists of three linear transformations, i.e., LayerNorm, ReLU activation, and Sigmoid. σ is the Sigmoid function to ensure that the output is within the range [0, 1].

2. Construct the key, value, and query of the attention mechanism.

Let $x_{ij}$ be the difference between the node features and the neighbor nodes features, which can be denoted as

(8) $$x_{ij}=\left[z_{i}^{1},z_{j}^{1}-z_{i}^{1}\right]$$

By layer normalization, query $q_{ij}$ can be obtained as

(9) $$q_{ij}=LN\left(x_{ij}\right)$$

Then, by using CAS-UNet scheme, we can calculate the key and value:

(10) $$\left(k_{ij},v_{ij}\right)=\text{CAS\_UNet}\left(x_{ij}\right)$$

CAS-UNet adopts an improved encoder-decoder architecture, where the encoder extracts node features through linear blocks and the decoder reconstructs these features and generates predictions. CAS-UNet promotes feature gating and fusion by introducing an Additive Attention Gate (AAG). Additionally, the model optimizes the interrelationship between feature channels through a cross-fusion channel attention module to enhance the effectiveness of fusion.

3. Calculate the attention coefficients. First, the model uses the scaled dot-product to calculate the unnormalized attention scores:

(11) $$\alpha_{ij}=\frac{q_{ij}^{⊤}k_{ij}}{\sqrt{L}}$$

where L is the length of the query vector.

Then, for each target node *i*, normalize over all neighboring nodes j using softmax:

(12) $${\bar{\alpha }}_{ij}=\frac{\exp\left(\alpha_{ij}\right)}{\sum_{j^{\prime }\in N\left(i\right)}\;\;\exp\left(\alpha_{ij^{\prime }}\right)}$$

Attention coefficient after applying Dropout ${\tilde{\alpha }}_{ij}$:

(13) $${\tilde{\alpha }}_{ij}=\mathrm{Dropout}\left({\bar{\alpha }}_{ij}\right)$$

4. Use the attention coefficients and dynamic edge weights to weight the values and construct the messages.

(14) $$m_{ij}=v_{ij}\cdot {\tilde{\alpha }}_{ij}\cdot e_{ij}$$

where ${\tilde{\alpha }}_{ij}$ is the attention coefficient after normalization and dropout, and $e_{ij}$ is the dynamic weight on the edge.

5. Use the Message Passing framework to aggregate information from neighbors:

(15) $$m_{i}=\sum_{j\in N\left(i\right)}\;m_{ij}$$

After obtaining the aggregated result $m_{i}$, a node’s own skip connection is also introduced, and the node’s intrinsic information is obtained through linear mapping:

(16) $$x_{r_{i}}=W_{skip\;}z_{i}^{1}+b_{skip}$$

where $W_{skip}$ is a weight matrix that represents the transformation from input features to output features, and $b_{skiip}$ is a bias vector that adjusts the output of the linear transformation.

6. Fusion of the β gating mechanism. The β gating mechanism provides an adaptive fusion strategy which dynamically adjusts the contribution of information by learning the gating parameter $\beta_{i}$:

(17) $$\beta_{i}=\sigma \left(f_{\beta }\left(\left[m_{i},x_{r_{i}},m_{i}-x_{r_{i}}\right]\right)\right)$$

where $f_{\beta }$ represents a learnable transformation function which generates an intermediate value based on different feature combinations of the input. This value is mapped to the range [0, 1] through a Sigmoid function, serving as the gating parameter for the fusion of the skip connection and neighbor aggregation information.

The final output of the module is $z_{i}^{2}$:

(18) $$z_{i}^{2}=\beta_{i}x_{r_{i}}+\left(1-\beta_{i}\right)m_{i}$$

The output $z_{i}^{2}$ undergoes a self-attention layer and a series of residual operations, resulting in the final output $z_{i}^{2\prime }$:

(19) $$z_{i}^{2\prime }=ReLU({(ReLU(BN(z_{i}^{2}+z_{attention}^{2})))+z}_{i}^{1})$$

where ReLU is the activation function, BN refers to the batch normalization layer, and $z_{attention}^{2}$ represents the features obtained from $z_{i}^{2}$ after passing through a self-attention layer.

### 3.2. Gated Multi-Head Attention Mechanism

Calculate the attention coefficient $\alpha_{ij}^{l}$:

(20) $$\alpha_{ij}^{l}=softmax\left(\frac{\left({Dec}_{k}^{i}W_{Q}\right){\left({Dec}_{k}^{j}W_{K}\right)}^{T}}{\sqrt{d_{h}}}\right)$$

where ${Dec}_{k}^{i}$ represents the input features of node i used for attention computation in the current module, $W_{Q}$ denotes the transformation matrix for the query, which converts the features of node  i  into a query vector, ${Dec}_{k}^{j}$ represents the input features of the neighboring node  j used for attention computation, $W_{K}$ denotes the transformation matrix for the key, and $d_{h}$ represents the vector dimension for each attention head, used to scale the dot-product attention. Softmax is an activation function that normalizes a vector of values into a probability distribution, where the sum of all probabilities equals 1.

The gating vector g can be calculated as

(21) $$g=tanh\left(W_{g}z_{i}^{2\prime }+b_{g}\right)$$

where $W_{g}\;$ denotes the weight matrix used to compute the gating vector, and $b_{g}$ represents the bias term used to adjust the output of the linear transformation.

The fused information is used to update the node information:

(22) $$z_{i}^{3}=z_{i}^{2\prime }+scale\times g⊙\left(\sum_{j\in N_{i}}\;\alpha_{ij}^{l}{Dec}_{v}^{j}W_{V}\right)$$

where $W_{V}\;$ is the value transformation matrix, scale represents the scaling factor, set to 0.1 in this paper, and ⊙  denotes the Hadamard product.

### 3.3. Dynamic Edge Enhancement

This layer dynamically generates edge features based on the current node features to enhance edge information. It also computes the updated edge values (edge features) using node features while simultaneously adjusting the node features.

1. Calculate the edge feature $e_{ij}$:

(23) $$e_{ij}=\left[x_{i},x_{j}\right]\in R^{2D}$$

where $x_{i}\;$ represents the source node features, and $x_{j}\;$ represents the target node features.

2. Map the concatenated features into the node-dimensional space using an MLP. Let $e_{ij}^{\prime }$ be the mapped edge feature:

(24) $$e_{ij}^{\prime }=BN\left(W_{e}e_{ij}+b_{e}\right)$$

where $W_{e}$ is the weight matrix, and $b_{e}$ is the bias vector, used to adjust the offset of the mapped features. BN refers to batch normalization.

3. Calculate the average of the edge features of each node’s neighbors. For each node i, the average edge feature of its neighboring nodes is denoted as ${\overset{ˆ}{e}}_{i}$:
  (25) $${\hat{e}}_{i}=\frac{1}{\left|N_{i}\right|}\sum_{j\in N_{i}}\;e_{ji}^{\prime }$$
  where $N_{i}$ is the set of neighboring nodes of node  i, indicating all the neighboring nodes connected to node  i, $\left|N_{i}\right|$ denotes the number of neighboring nodes, and $e_{ji}^{\prime }$ is the edge feature mapping value from neighboring node j to node  i.

4. By linear transformation and normalization on the current node features, we can obtain the updated intrinsic feature $x_{i}^{\mathrm{transformed}}$
  (26) $$x_{i}^{transformed}=BN\left(W_{n}z_{i}^{3}+b_{n}\right)$$
  where $\;W_{n}$ is the weight matrix, and bnb_nbn is the bias vector.

5. By integrating the edge features into the node features, the final node feature $z_{i}^{4}$ can be obtained as

(27) $$z_{i}^{4}=x_{i}^{transformed}+{\hat{e}}_{i}$$

The CAS-Unet model adopts a hierarchical update strategy, alternating between edge enhancement and gated attention every two layers. The final control command is constrained by a dynamic range. This architecture employs CAS-UNet for multi-scale feature extraction, with the gating mechanism stabilizing the training process. The dynamic edge weights adaptively adjust the interaction intensity. The entire process can be simplified as:

(28) $$z_{i}^{4}=f_{edge\;enhance}\left(f_{g}\left(f_{conv1}\left(f_{linear0}\left(x_{i}\right),E\right)\right),E\right)$$

where $z_{i}^{4}$ represents the node features after passing through all the previous linear layers, graph convolutional layers, and the gated attention and dynamic edge enhancement added at appropriate levels.

The final output is the vehicle control command:

(29) $$u_{i}=\tanh\left(W_{3}\left(conv2(z_{i}^{4})\right)+b_{3}\right)$$

where conv2 is the process of graph convolutional layer in Section 3.1, $W_{3}$ and $b_{3}$ are the coefficients of the final linear mapping layer, and tanh is the activation function.

## 4. Simulation

We create a simulation environment with multiple vehicles and obstacles to evaluate the CAS-GNN model we proposed. In the simulation, we assess its trajectory efficiency and planning success rate for path planning, as well as its obstacle avoidance performance.

### 4.1. Definition of the Dataset

The initial positions of the vehicles are distributed within a ±15 m range centered around the origin. The target point is located in the symmetric direction relative to the center of the initial positions, and the speed follows a normal distribution denoted as N(2.5, 5). Obstacles are randomly distributed within ±7 m on both sides of the vehicle’s driving path. Considering that the radii of these obstacles range from 1 to 3 m, and that the actual minimum distance between the vehicle and any obstacle is approximately 1 meter, it is required that obstacles be situated at least 5 m away from both start and end points. The input data for vehicles includes their starting position, starting angle, speed, and end position. Obstacle data encompasses their position and radius.

The dataset used for training is a simulation dataset, including 1–3 vehicles and 0–4 static obstacles. A total of approximately 20,961 trajectories were generated, with each trajectory consisting of 120 time steps. These labels were generated via the cost function presented in formula 2–5. By optimizing these objectives, the generated control commands are utilized as the labels for the model. The generated label data is partitioned into a training set and a validation set, with the ratio of the training set to the validation set being 4:1. Since that obstacles with various shapes can be assembled with using circular obstacles of different sizes, this experiment focuses on circular obstacles case.

### 4.2. CAS-GNN Training Details and Simulation Environment Setup

The training dataset for the CAS-GNN model is constructed as follows. We construct the multi-vehicle and multi-obstacle scene data, then we generate the environment information matrix and output control input-output pairs by data augmentation. The initial learning rate is set to 0.001. In the case that the validation loss does not decrease for 10 epochs, the learning rate is multiplied by 0.2. The minimum of learning rate is set to 0.00000001. The weight decay coefficient is set to 0.000005, the batch size is set to 4096, and early stopping is employed (training is stopped after 15 epochs without improvement). During the training procedure, the loss function computes the weighted mean square error (MSE) between the predicted value and the true value. Specifically, different weights are assigned to the error of each control command in accordance with the time step. Generally speaking, a higher weight is assigned to the earlier time step. In the presence of static obstacles, an additional loss term is calculated and weighted before being incorporated into the total loss. The final loss function yields the weighted total loss, and the model is trained by minimizing this loss value. The hardware configuration is shown in Table 1:

The following three metrics, which can evaluate navigation accuracy, safety, and path quality, are used to assess the performance of the proposed scheme.

(PPSR) Path Planning success rate: The planning success rate is the proportion of collision-free arrivals that meet endpoint position deviation ≤ 2 m and heading angle deviation ≤ 0.1 radians, obtained by dividing the number of successfully planned scenarios by the total number of scenarios.
Collision rate: This refers to the number of vehicle-obstacle/vehicle-vehicle collisions per unit travel distance, with geometric collisions accurately detected using the Separating Axis Theorem, obtained by dividing the number of collision scenes by the total number of scenes.
Trajectory efficiency, which is the ratio of the ideal straight-line distance to the actual travel distance in successful cases. The trajectory efficiency reflects the optimality of the path.

### 4.3. Results

The path planning success rate (PPSR), collision rate, and trajectory efficiency of the proposed CAS-GNN model and the A-GNN model in various vehicle-obstacle scenarios are presented in Table 2.

Both models exhibit a decrease in PPSR as the number of obstacles or vehicles increases. However, when there is only one or two vehicles present, the PPSR of the CAS-GNN model is slightly lower than that of the A-GNN model. This discrepancy can be attributed to the inclusion of self-loop edges within the edge enhancement module of the CAS-GNN model, which becomes more pronounced with a smaller number of vehicles. Additionally, each vehicle has limited effective interaction relationships; thus, the multi-head mechanism may lead to dispersed attention. As the number of vehicles increases, it is observed that the CAS-GNN model achieves a better PPSR compared to the A-GNN model. Table 2 comparison of planning success rate, collision rate, and trajectory efficiency in scenarios with different obstacle densities.

Compared with the A-GNN model, the CAS-GNN model can obtain a lower collision rate and better trajectory efficiency.

**Figure 3:**
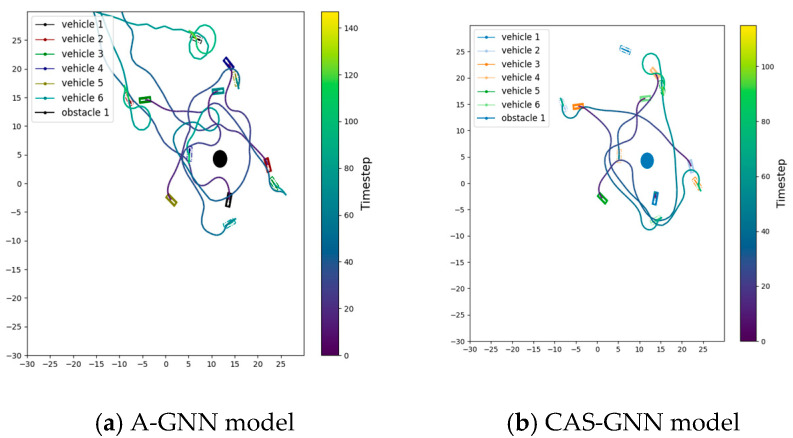
Trajectory graph of the A-GNN model and the proposed CAS-GNN. It can be clearly observed that the CAS-GNN model proposed in this paper outperforms the A-GNN model in terms of efficiency.

The trajectory graph of the two models with six vehicles and one obstacle is shown in Figure 3. As shown, the trajectory length of the CAS-GNN model is significantly shorter than that of the A-GNN model.

**Figure 4:**
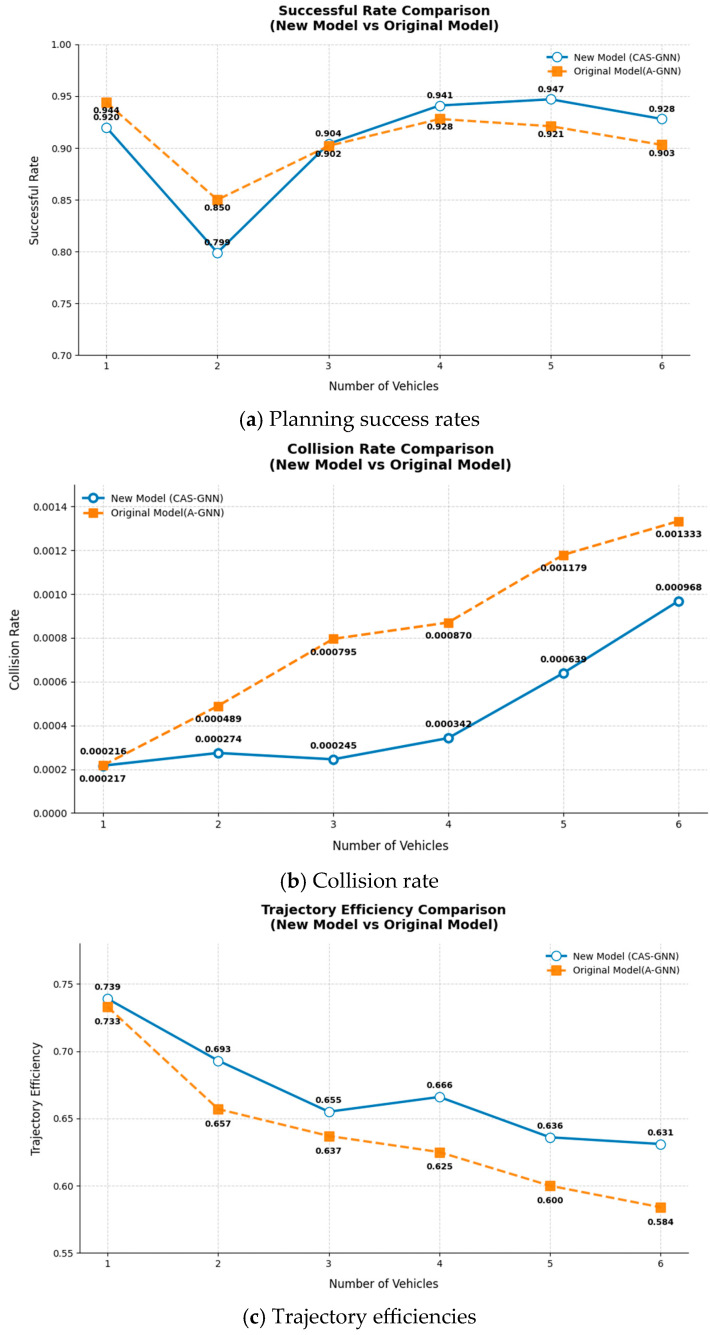
Comparisons of planning success rates, collision rates, and trajectory efficiencies between the CAS-GNN and A-GNN models with different numbers of vehicles.

In the context of varying numbers of vehicles, Figure 4 presents a comparison of planning success rates, collision rates, and trajectory efficiencies between the CAS-GNN and A-GNN models. For the scenario involving six vehicles, the CAS-GNN model demonstrates an increase in the planning success rate of 2.7%, a reduction in the collision rate of 23%, and an enhancement in trajectory efficiency of 8%. The proposed CAS-GNN model is capable of generating smooth and safe paths, showcasing remarkable generalization ability.

**Figure 5:**
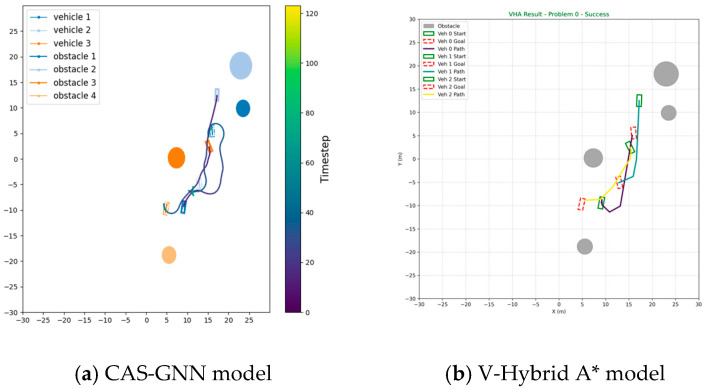
Trajectory graph of the V-Hybrid A* model and the proposed CAS-GNN model.

**Figure 6:**
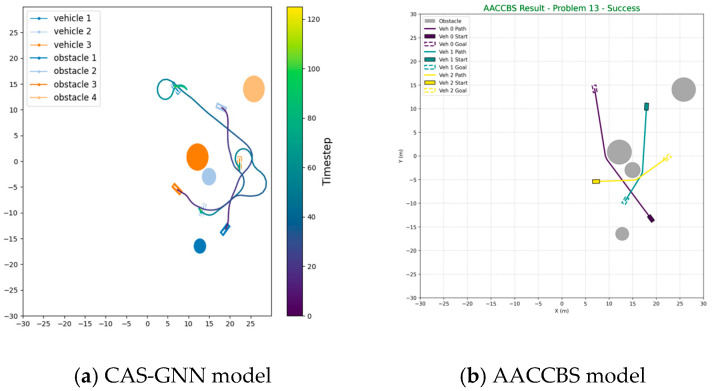
Trajectory graph of the AACCBS model and the proposed CAS-GNN model.

We now conduct a comparative analysis of our proposed CAS-GNN model against two traditional path-planning algorithms: Anytime A* Conflict-based Search (AACCBS) and V-Hybrid A*. This comparison is executed across 100 scenarios, each involving three vehicles and four obstacles. The paths generated by both AACCBS and V-Hybrid A* consist of discrete points; consequently, we implement an interpolation process for these paths in our simulation. The original straight-line trajectories produced by these two algorithms demonstrate high efficiency. However, following the interpolation process, the resulting paths exhibit a significantly elevated collision rate compared to that of our proposed algorithm. This discrepancy can be attributed to the fact that both AACCBS and V-Hybrid A* rely on search-based and optimization-based methods, which tend to perform inadequately in environments characterized by a high density of potential collisions. As illustrated in Table 3, the results clearly indicate that our proposed CAS-GNN algorithm surpasses both AACCBS and V-Hybrid A*. Figure 5 and Figure 6 presents the trajectories planned by all three models within a scenario featuring three vehicles and four obstacles.

## 5. Conclusions

In this paper, we propose the CAS-GNN model, which integrates CAS-UNet with a graph neural network (GNN). The CAS-GNN model uses an attention-based graph neural network to construct a multi-vehicle interaction framework, facilitating distributed decision-making through the aggregation of global and local information. Furthermore, CAS-UNet efficiently extracts environmental features. Its lightweight architecture and residual connection design significantly surpass those of traditional U-Net models. Simulation results demonstrate that this model achieves a high planning success rate, enhanced trajectory efficiency, and a low collision rate across scenarios characterized by varying numbers of obstacles and multiple vehicles. Additionally, it exhibits strong generalization capabilities, enabling adaptation to diverse environmental conditions. This effectively enhances the path planning abilities of multiple autonomous vehicles operating in environments with both multiple vehicles and obstacles, thereby improving obstacle avoidance accuracy and robustness. It is important to note that the analysis conducted in this study was limited to scenarios involving homogeneous circular obstacles. Future research could extend the application of this algorithm to environments featuring heterogeneous shapes and dynamic obstacles. Moreover, subsequent investigations may explore the performance of the model by incorporating vehicle dynamics models.

## Figures and Tables

**Table 1:** The configuration of the hardware environment where the experiment is located.

Configuration	Parameter
OS	Windows10 64-bit
CPU	Intel(R)Core(TM)i9-14900KF, Santa Clara, CA, USA
GPU	NVIDIARTX4080, NVIDIA, Santa Clara, CA, USA
Video memory	64 GB
GPU-accelerated environment	CUDA11.8
Network architecture	PyTorch 2.1.2
Programming	Python 3.8

**Table 2:** The path planning success rate (PPSR), collision rate, and trajectory efficiency of the CAS-GNN model and the A-GNN model in various vehicle-obstacle scenarios.

		**Planning Success Rate**		**Collision Rate**		**Trajectory Efficiency**	
**Number of Vehicles**	**Number of Obstacles**	**Proposed CAS-GNN**	**A-GNN**	**Proposed CAS-GNN**	**A-GNN**	**Proposed CAS-GNN**	**A-GNN**
1	0	1.000	1.000	0	0	0.8016	0.7845
1	1	0.959	0.970	0	0	0.7630	0.7625
1	2	0.949	0.949	0	0.000522	0.7399	0.7197
1	3	0.870	0.939	0.000283	0.000267	0.7060	0.7094
1	4	0.819	0.860	0	0.000297	0.6845	0.6921
2	0	1.000	1.000	0	0	0.7460	0.7429
2	1	0.9449	0.959	0	0	0.7204	0.6834
2	2	0.7950	0.855	0.000618	0.000372	0.6800	0.6432
2	3	0.6600	0.755	0.000223	0.001454	0.6392	0.6128
2	4	0.5950	0.680	0.000523	0.000611	0.6782	0.6023
3	0	0.9966	0.993	0	0.000112	0.7180	0.6805
3	1	0.9566	0.943	0.000117	0.000775	0.7100	0.6851
3	2	0.9133	0.893	0.000117	0.000864	0.6598	0.6325
3	3	0.8566	0.889	0.000209	0.001098	0.6190	0.6038
3	4	0.7966	0.790	0.000117	0.001729	0.6204	0.5854
4	0	0.9925	0.965	0.000081	0.000532	0.7204	0.6758
4	1	0.9624	0.973	0.000155	0.000363	0.6612	0.6300
4	2	0.9024	0.902	0.000396	0.000932	0.6569	0.6246
4	3	0.9075	0.873	0.000689	0.001650	0.6223	0.5696
5	0	0.9819	0.966	0.000231	0.000389	0.6497	0.6235
5	1	0.9359	0.910	0.000663	0.001459	0.6429	0.6000
5	2	0.9219	0.888	0.001022	0.001678	0.6171	0.5775
6	0	0.9683	0.942	0.000546	0.000823	0.6433	0.5996
6	1	0.9300	0.895	0.000836	0.001386	0.6427	0.5849
6	2	0.8866	0.872	0.001422	0.001789	0.6070	0.5683

**Table 3:** Comparison of the performance of three models.

	Success Rate	Collision Rate	Trajectory Efficiency
CAS-GNN	1.0000	0.000117	0.6204
V-Hybrid A*	0.9400	0.71	0.8854
AACCBS	0.4700	0.3900	0.98

## Data Availability

The data is unavailable due to privacy and ethical restrictions.
